# Screening for Myocardial Injury after Mild SARS-CoV-2 Infection with Advanced Transthoracic Echocardiography Modalities

**DOI:** 10.3390/diagnostics12081941

**Published:** 2022-08-11

**Authors:** Gergely Rácz, Hedvig Takács, Árpád Kormányos, Bianka Polestyuk, János Borbás, Nándor Gyenes, Noémi Schvartz, Gergely Németh, Zsigmond Tamás Kincses, Róbert Sepp, Viktória Nagy

**Affiliations:** 1 Division of Non-Invasive Cardiology, Department of Internal Medicine, University of Szeged, 6725 Szeged, Hungary; 2 Department of Radiology, University of Szeged, 6725 Szeged, Hungary

**Keywords:** SARS-CoV-2, myocardial injury, echocardiography, myocardial work

## Abstract

Although the clinical manifestations of SARS-CoV-2 viral infection affect mainly the respiratory system, cardiac complications are common and are associated with increased morbidity and mortality. While echocardiographic alterations indicating myocardial involvement are widely reported in patients hospitalized for acute COVID-19 infection, much fewer data available in non-hospitalized, mildly symptomatic COVID-19 patients. In our work, we aimed to investigate subclinical cardiac alterations characterized by parameters provided by advanced echocardiographic techniques following mild SARS-CoV-2 viral infection. A total of 86 patients (30 males, age: 39.5 ± 13.0 yrs) were assessed 59 ± 33 days after mild SARS-CoV-2 viral infection (requiring no hospital or <5 days in-hospital treatment) by advanced echocardiographic examination including 2-dimensional (2D) speckle tracking echocardiography and non-invasive myocardial work analysis, and were compared to an age-and sex-matched control group. Altogether, variables from eleven echocardiographic categories representing morphological or functional echocardiographic parameters showed statistical difference between the post-COVID patient group and the control group. The magnitude of change was subtle or mild in the case of these parameters, ranging from 1–11.7% of relative change. Among the parameters, global longitudinal strain [−20.3 (−21.1–−19.0) vs. −19.1 (−20.4–−17.6) %; *p* = 0.0007], global myocardial work index [1975 (1789–2105) vs. 1829 (1656–2057) Hgmm%; *p* = 0.007] and right ventricular free wall strain values (−26.6 ± 3.80 vs. −23.8 ± 4.0%; *p* = 0.0003) showed the most significant differences between the two groups. Subclinical cardiac alterations are present following even mild SARS-CoV-2 viral infection. These more subtle alterations are difficult to detect by routine echocardiography. Extended protocols, involving speckle-tracking echocardiography, non-invasive measurement of cardiac hemodynamics, and possibly myocardial work are necessary for detection and adequate follow-up.

## 1. Introduction

Coronavirus disease 2019 (COVID-19), caused by the virus severe acute respiratory syndrome-coronavirus-2 (SARS-CoV-2) is an ongoing pandemic with—as of 22 May 2022—over 522 million cases and over 6 million deaths reported. While the disease most noticeably manifests as a respiratory infection it can cause a number of cardiac complications, which are not only common, affecting as much as 20–25% of those infected, but are major contributor of disease burden and mortality as noted in several studies [1,2]. This highlights the need to consider COVID-19 as a multi-system disease with an important focus on the circulatory system.

The involvement of the cardiovascular system has been proven in all stages of the illness, although exact pathomechanisms and incidence remains uncertain. Cardiac involvement of COVID-19 infection may be due to multiple factors, the most important ones include myocardial damage due to acute systemic inflammatory response; hypoxia secondary to acute respiratory failure; microvascular and macrovascular thrombosis due to systemic inflammation and endothelial dysfunction; and possibly direct viral infection of the myocardium [3]. The most common forms of cardiac involvement were reported as myocardial damage, cardiac failure, acute coronary syndrome, and thromboembolic episodes [3]. Cases of fulminant myocarditis with both atrial and ventricular arrhythmias have also been previously described in the setting of COVID-19 [4].

It is of major importance to investigate the degree of residual cardiac involvement several weeks or months after recovery, due to the very high number of patients affected. This is especially true for patients with mild COVID-19, not hospitalized for the disease. Several cardiac magnetic resonance (CMR) studies have shown that, independent of overall course of the acute illness, a large part of patients showed signs of ongoing inflammation, oedema, fibrosis, and decreased functional parameters [5,6,7,8]. As cardiac MR has limited accessibility, especially for follow-up studies, two-dimensional (2D) echocardiography is the most preferred imaging modality for the assessment of most cardiovascular diseases. Among echocardiographic modalities, speckle tracking echocardiography (STE) has emerged as an echocardiographic technique providing novel parameters for the evaluation for myocardial function. The latter includes strain parameters and myocardial work parameters, that evaluates LV work estimated by employing blood pressure and left ventricular global longitudinal strain (GLS) [9]. These parameters are more sensitive for predicting left ventricular myocardial injury and future cardiac events [10].

Echocardiographic alterations indicating myocardial involvement of the heart are frequent and widely reported in patients hospitalized for acute COVID-19 infection [11,12]. On the contrary, there are much fewer data in non-hospitalized, mildly symptomatic COVID-19 patients, especially with regard to advanced echocardiographic parameters. Therefore, in our study we aimed to address whether cardiac alterations, characterized by parameters provided by advanced echocardiographic techniques, e.g., strain and myocardial work, are present in patients recovered from mild COVID-19 infection. Here, we present results analyzing otherwise healthy and young patients presenting weeks after recovery from COVID-19 infection with persisting fatigue, exercise intolerance, and tachycardia, who underwent advanced echocardiographic examination.

## 2. Patients and Methods

### 2.1. Patient Population

Patients recovered from mild COVID-19 infection (defined as not requiring hospital treatment or requiring <5 days hospital treatment) and having residual symptoms were entered into the study. Initially 102 patients were assessed because of residual symptoms such as chronic fatigue, difficulty of carrying out previously undemanding physical activity, and palpitations. Out of the initially assessed subjects, 16 patients were ruled out due to suboptimal image quality, known diabetes and previously known coronary artery disease. 

Of the remaining 86 patients [30 (34.9%) males, avg. age: 39.5 ± 13.0 yrs (age range: 13–67 yrs; 90% of patients and 77% of the patients being <55 and <50 yrs old, respectively)] a few had well controlled hypertension, and 1 patient had mixed connective tissue disease which was not active immunologically at the time of examination. Most patients had mild symptoms during their acute illness with COVID-19, with only 4 patients requiring short (<5 days) hospitalization for moderate symptoms, none having troponin T elevation or requiring intensive care unit treatment. The number of patients receiving any type of specific anti-viral treatment was negligible, with 2 patients having received remdesivir, and 1 patient having received favipiravir.

At the time of assessment (59 ± 33 days after COVID-19 diagnosis; 84% and 90% of the patients were examined within 93 and 100 days, respectively), no patient had elevated troponin T levels or >200 pg/mL NTproBNP levels. No major ECG changes were detected in the patients apart of >100 bpm sinus tachycardia which was present in 2 patients.

An age- and sex-matched group of 60 ostensibly healthy subjects [24 (40.0%) males, avg. age: 40.3 ± 11.0 yrs] served as a control group. None of the subjects had a history of any illness or was on any medication. The control group either did not have COVID-19 infection or had COVID-19 infection >1 year apart of the examination. The baseline clinical characteristics of the study and control patients did not differ statistically (Table 1).

### 2.2. Echocardiographic Protocol

Blood pressure was measured at rest in a sitting position immediately prior to the echocardiographic exam.

All patients underwent advanced echocardiographic examination including 2-dimensional (2D) speckle tracking echocardiography for the left and right ventricles and the left atrium, and non-invasive myocardial work analysis. All measurements included in this study were assisted or gated with electrocardiogram. Standard measurements of dimensions of the left- and right-side of the heart were carried out with indexing for body surface area (BSA) where necessary. Systolic function of the left ventricle was thoroughly measured, with ejection fraction according to the biplane Simpson method, and hemodynamic parameters derived from Doppler measurement of the left ventricular outflow tract (LVOT) velocity time integral (VTI) and the size of the LVOT, and well as the resting heart rate. Examination of the diastolic function was performed according to the latest guidelines and thus including tissue velocity imaging (TVI). The investigation of the systolic function of the right ventricle included tricuspid annular plane systolic excursion, and the peak systolic velocity of the tricuspid annulus measured by TVI. Speckle-tracking strain analysis of the left-side of the heart included measurement of global longitudinal strain (GLS) from apical 2-, 3-, and 4-chamber views, and derived global myocardial work parameters: global work index (GWI), global constructive work (GCW), global wasted work (GWW) and global work efficiency (GWE). The right ventricular longitudinal free wall strain was also measured with the dedicated right ventricular speckle-tracking software. The regional strain and myocardial work differences were not investigated in this study. All examinations were carried out with a GE Vivid E95 R3 (General Electric Healthcare) cardiac ultrasound system.

### 2.3. Statistical Analysis

Continuous variables were expressed as mean ± standard deviation (SD) or median (interquartile range, IQR). The Kolmogorov–Smirnov test was used for testing the normality of the distribution of continuous variables. Differences between groups were analyzed with the Student’s T test, in case of normally distributed continuous variables, and with the Mann–Whitney U test, in case of non-normally distributed continuous variables. The Chi-square and Fisher’s exact tests were used for categorical variables. The Pearson’s or Spearman’s correlation analysis was used to analyze correlations between continuous variables. We performed multivariable linear regression analyses to examine the independent correlates between GLS and myocardial work parameters and standard and advanced echocardiographic parameters.

To characterize the magnitude of changes, relative difference regarding parameters between the study and the control groups were calculated and were expressed as the relative percentage difference between the median of the parameters (to exclude the effect of outlier values).

Statistical analysis was conducted with MedCalc^®^ Statistical Software version 20.106 (MedCalc Software Ltd., Ostend, Belgium; https://www.medcalc.org; 2022). A *p* < 0.05 value was considered as statistically significant.

## 3. Results

Altogether, variables from eleven echocardiographic categories representing morphological or functional echocardiographic parameters showed statistical difference between the post-COVID patient group and the control group. The magnitude of change was subtle or mild in case of these parameters, ranging from 1–11.7% of relative change (either increase or decrease in the parameter). Detailed comparison of the echocardiographic parameters regarding dimensions and function of the left-and right-side of the heart is given in Table 2, Table 3 and Table 4.

### 3.1. Dimensional Parameters of the Left-Side of the Heart

Among parameters representing dimensions and volumes of the left-side of the heart, the LV end diastolic diameter (46.2 ± 4.2 vs. 47.9 ± 4.2 mm; *p* = 0.020), the LV end systolic volume index [15.5 (13.3–18.6) vs. 17.1 (15.2–21.0) mL/m^2^; *p* = 0.013] and the LV posterior wall thickness [8.5 (8.0–9.0) vs. 9.0 (8.0–10.0) mm; *p* = 0.042] showed significant difference between the post-COVID and the control group (Table 2). However, the relative difference was <10% in the case of all the different parameters, indicating only a mild dilatation of the LV in the post-COVID group.

### 3.2. Functional Parameters of the Left-Side of the Heart

Parameters representing the systolic function of the LV, including LV ejection fraction [68.0 (65.0–70.0) vs. 66.0 (60.0–70.0)%; *p* = 0.031], stroke volume [75.5 (70.0–87.0) vs. 70.5 (61.0–78.0) mL; *p* = 0.004] and stroke volume index [41.6 (38.9–43.7) vs. 37.4 (33.5–41.8) mL/m^2^; *p* = 0.0003] were all significantly, but mildly decreased in the post-COVID patient group (Table 3). Here again, the relative decrease in these parameters was 10% the most. Interestingly, despite the mild decrease in stroke volume, cardiac output and cardiac index were not different between the groups, as heart rate was significantly increased in post-COVID patients (70.9 ± 10.8 vs. 75.6 ± 13.4 bpm; *p* = 0.029) presumably compensating for the decrease in stroke volume.

**Figure 1:**
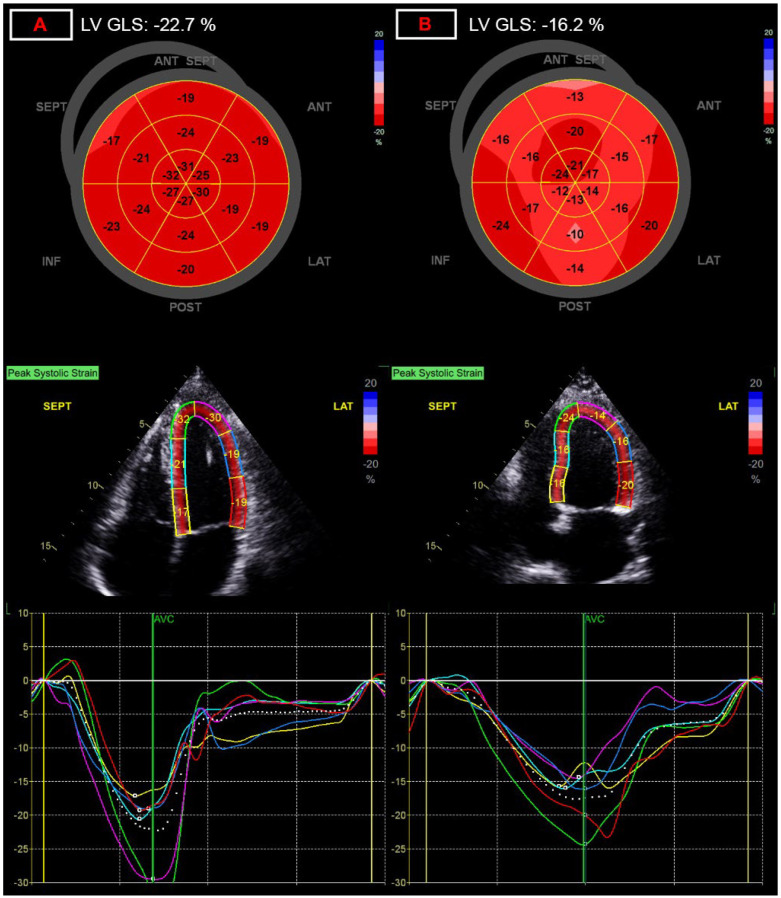
Representation of alterations of global longitudinal strain (GLS) measurement. (**A**) panel: normal left ventricular global longitudinal strain of −22.7%; (**B**) panel: decreased left ventricular global longitudinal strain of −16.2%, after COVID-19 infection.

Among parameters representing contractile function of the LV, global longitudinal strain showed one of the most significant differences between the two groups [−20.3 (−21.1–−19.0) vs. −19.1 (−20.4–−17.6)%; *p* = 0.0007], with a relative decrease of 5.9% (Figure 1). The decreased GLS values correlated with many parameters of LV dimension and function in univariate correlation analysis (Table 5) but correlated only with LV stroke volume index (partial correlation coefficient, r_partial_: −0.284; *p* = 0.029), and the left atrial volume index (r_partial_: −0.343; *p* = 0.008) in the multivariate regression analysis.

Parameters representing LV diastolic function did not differ between the study and the control group.

### 3.3. Myocardial Work Parameters

**Figure 2:**
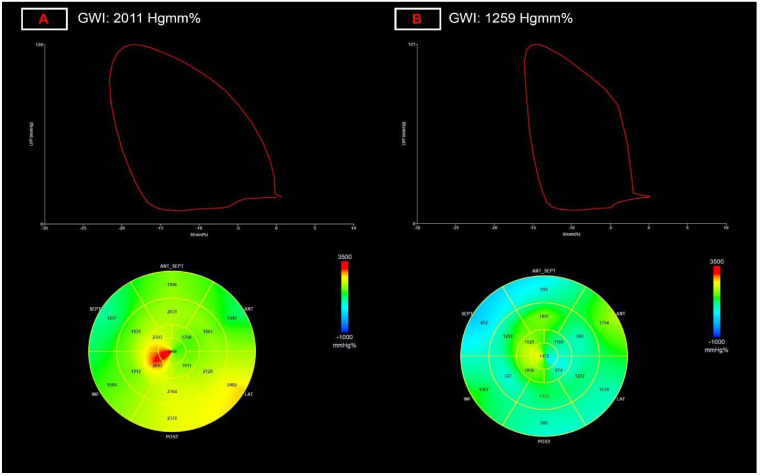
Representation of alterations global myocardial work index (GWI) measurement. (**A**) panel: normal left ventricular myocardial work index of 2011 Hgmm%; (**B**) panel: decreased left ventricular myocardial work index of 1259 Hgmm%, after COVID-19 infection.

With regard to myocardial work parameters, global myocardial work index (GWI) values [1975 (1789–2105) vs. 1829 (1656–2057) Hgmm%; *p* = 0.007; Figure 2] and global work efficiency (GWE) values [96 (94–97) vs. 95 (93–96)%; *p* = 0.038] were significantly decreased, and the other two myocardial work parameters, LV global constructive work [2383 (2226–2577) vs. 2341 (2094–2559) Hgmm%; *p* = 0.080] and LV global wasted work [99 (63–129) vs. 107 (77–151) Hgmm%; *p* = 0.088] also showed marked differences, close to significancy (Table 3). The decreased GWI and GWE values correlated with many parameters of LV dimension and function in univariate correlation analysis (Table 5) but correlated with none of the parameters in the multivariate regression analysis (apart of GLS and systolic RR which they are derived from).

### 3.4. Dimensional and Functional Parameters of the Right-Side of the Heart

**Figure 3:**
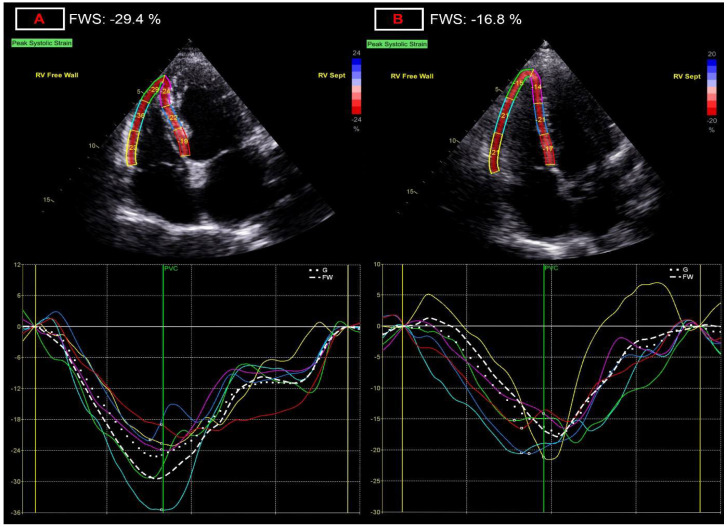
Representation of alterations of right ventricular free wall strain (FWS) measurement. (**A**) panel: normal right ventricular free wall strain of −29.4%; (**B**) panel: decreased right ventricular free wall strain of −16.8%, after COVID-19 infection.

Dimensions of the right heart did not show statistical difference between the two groups. Among functional parameters, tricuspid annular plane systolic excursion values were significantly decreased in post-COVID patients (23.75 ± 2.8 vs. 22.5 ± 3.4 mm; *p* = 0.039), while tricuspid annular s’ velocity values were similar. However, right ventricular free wall strain values (−26.6 ± 3.80 vs. −23.8 ± 4.0%; *p* = 0.0003; Figure 3) were significantly decreased in post-COVID patients, showing the most significant change, and showing the largest relative difference between the two groups at 11.7% (Table 4).

### 3.5. Valvular Alterations

No hemodynamically significant stenotic valvular disease has been found in either group. Mild aortic (4 patients, 4.65%), mitral (13 patients, 15.1%), pulmonary (28 patients, 32.6%), tricuspid (8 patients, 9.3%) regurgitation was found (data not shown), however, we considered all these hemodynamically not significant.

## 4. Discussion

Although echocardiographic alterations in acutely ill patients with COVID-19 infections are well characterized [11,12], there are still few data regarding the long-term cardiac consequences of the disease, especially in the young and affected by a mild form of the disease. In our study we provide data that subclinical cardiac alterations, characterized by parameters provided by advanced echocardiographic techniques, are frequent following mild SARS-CoV-2 viral infection. This subclinical myocardial injury after mild SARS-CoV-2 infection cannot be detected with laboratory tests, ECG or standard LV echocardiography parameters, however, advanced echocardiographic modalities may provide parameters, such as global longitudinal strain or myocardial work parameters, that indicate subtle LV or RV functional injury.

The occurrence of cardiac alterations is an important aspect of COVID-19 infection. Cardiac involvement due to COVID-19 infection is thought to be multifactorial; that includes myocardial damage due to acute systemic inflammatory response; hypoxia secondary to acute respiratory failure; microvascular and macrovascular thrombosis due to systemic inflammation and endothelial dysfunction; and possibly direct viral infection of the myocardium [13]. Multiple autopsy studies showed that viral presence with active inflammation, and even myocardial inflammatory storm is often present, along with endothelial damage and microthrombi. It is generally hypothesized that both a direct organ damage, and a secondary damage due to the inflammatory response plays a role in cardiac involvement [14]. Pellegirini et al. reported that the most common cause for cardiomyocyte necrosis appears to be of thrombotic origin in SARS-CoV-2 infection, microthrombi being by far the most common [15]. This is caused by direct endothelial infection through ACE2 receptors, but perhaps more importantly secondary to endothelial activation caused by excessive immune system activation. This hyperinflammatory state plays a major role in the course of the infection and its pulmonary involvement, but its cardiac effect must be equally emphasized [16]. Especially this pathomechanism can lead to severe illness in both children (Multisystem Inflammatory Syndrome in Children, MIS-C) and adults (MIS-A) weeks after initial infection [17]. Both NT-proBNP and hs-Troponin has been described as an independent predictor for adverse outcome in patients requiring hospitalization. Importantly in the case of NT-proBNP, this appears to be unrelated to the development of acute heart failure [18]. Elevated troponin levels on admission were similarly found to be associated with increased 30-day mortality. Interestingly such risk was more robustly predicted in less severe waves of the pandemic [19].

Echocardiographic alterations indicating myocardial involvement of the left- or right-side of the heart are frequent and widely reported in patients hospitalized for acute COVID-19 infection [11,12,20]. These alterations include measures of left ventricular systolic and diastolic function, multiple parameters of right ventricular systolic performance as well as pulmonary artery flow acceleration time. In several studies, decreased LVEF was found to be associated with clinical deterioration and mortality [21,22]. Elevated NT-proBNP and troponin levels were predictive of reduced stroke volume, cardiac output, and cardiac index, which were in turn associated with adverse outcome [22]. However, in contrast to non-invasive hemodynamics, elevation of troponin-I and reduction in LVEF were not significantly related. Not only systolic but diastolic function of the left ventricle is affected, and elevated E/e’ is independently associated with mortality [22]. Remarkably, impaired LV global longitudinal strain is not only associated with increased mortality, but a cut of value of ≤15.20% was even showed to have a predictive value with a sensitivity of 77% and a specificity of 75% [23]. Janus et al. also demonstrated that a reduction in GLS is a powerful predictor of mortality in COVID-19 patients [24].

On the contrary to the above findings in hospitalized patients, data on echocardiographic changes in patients with mild (requiring no hospitalization) COVID-19 infection are scarce. Studies have shown that absolute value of left ventricular global longitudinal strain is lower in patients suffering from mild COVID-19 symptoms on initial evaluation, without significant difference in more traditional parameters compared to a healthy control group [25]. In a preliminary report, Uzieblo-Zyczkowska et al. found no difference in GLS after mild COVID infection in post-COVID patients and controls, although assessing only 31 patients [26]. It is reported that LV GLS has some value in detecting subclinical left ventricular dysfunction in patients recovered from COVID-19 even in cases of asymptomatic or mild illness, but notably, the parameter was less robust compared to those who had severe illness [27]. In a prospective, observational study of Ikonomidis et al. assessing 70 COVID-19 patients (34.28% with mild disease) 12 months post-infection, GLS values in COVID-19 patients showed a borderline improvement compared to values at 4 months, though these remained impaired compared to controls [28]. Our data also supports the observation that GLS is the parameter which shows one of the most significant differences in the post-COVID group. However, these changes are minor (~6% relative change) and are difficult to utilize on a single patient basis since many patients fall into the “normal” range. In another important study, 383 patients were screened for cardiac involvement in the post-acute phase of COVID-19 [29]. Approximately a quarter of the patients (*n* = 102) had some sort of cardiac sequelae, including left ventricular systolic and diastolic dysfunction, increased pulmonary arterial pressure and pericardial disease, however, most had moderate pulmonary involvement initially. The authors found that during follow-up the number of patients with any abnormality steadily decreased and the remaining showed less severe alterations. It is also important to note that this patient population was enrolled in three different waves of the pandemic, and that according to the authors’ conclusions differing viral strains showed different patterns.

Our results showed that apart of GLS, myocardial work (MW) parameters were the ones that was most significantly altered in the post-COVID group. Although GLS is still a relatively new, well-validated tool for the evaluation of cardiac alterations, its clinical performance is influenced by its dependency on changes in ventricular load. On the other hand, LV MW is a novel parameter, based on the same speckle tracking-based method which eliminates some of the load dependency of GLS. LV MW estimates LV work by employing brachial artery blood pressure and LV GLS. An estimated LV pressure is calculated using an empiric reference pressure curve which was established by using invasive pressure measurements from a number of patients with different pathologies and was further adjusted to the duration of different phases of the cardiac cycle as determined by echocardiography [9]. Even though MW still possesses a few limitations, it has been found to be a more sensitive index of segmental and global LV performance compared to EF and GLS. The additive value of detecting MW alteration has been shown for many cardiac diseases including cardiac dyssynchrony, heart failure, cardiomyopathies, coronary artery disease and valvular heart disease [9]. With regard to COVID-19, significantly reduced GWI has been first demonstrated in a COVID-19 positive patient who had normal EF and GLS parameters on admission which showed marked improvement after one month [30]. In the study of Ikonomidis et al., the authors found that, when examined at 4 months after infection, COVID-19 patient showed significantly worse myocardial work efficiency and higher degree of wasted work compared to control group. Furthermore, their findings showed that at 12 months, there was some relevant improvement of these values; however, these markers remained impaired compared to controls [28]. In a retrospective cohort of 136 patients hospitalized for COVID-19, 79% of patients had abnormal MWE despite 81% had normal left ventricular ejection fraction. Higher MWE was associated with lower in-hospital mortality, in addition, increased systemic inflammation measured by interleukin-6 level was associated with reduced MWE [31].

The impact of SARS-CoV-2 infection on the right ventricle was among the first cardiac phenomena described. In our study, parameters of right ventricular systolic function and contractility, TAPSE and RV free ventricular strain was impaired in post-COVID patients. The involvement of the right ventricle is thought to be due to the increase in afterload secondary to increases in pulmonary vasculatory resistance caused by pulmonary inflammation, ARDS or pulmonary embolism/thrombosis. RV dysfunction may also be caused by direct myocardial damage by SARS-CoV-2, endothelitis, due to microvascular and macrovascular dysfunction, overload of vasoactive peptides, and inflammatory injury. As our patient group did not require any or prolonged hospitalization due to respiratory complications the latter mechanisms seem to be prominent in explaining the RV impairment in our patient group. Similarly to our findings, others have reported the value of RV strain in detection of long-term persisting right ventricular involvement, appearing to be one of the strongest predictors. The correlation of RV strain values and inflammatory markers also suggest that the immune response plays a decisive role in cardiac involvement [32].

As for cardiac MRI, both a recent state-of-the-art review, and a large meta-analysis highlight that CMR is a highly sensitive imaging tool for cardiac alterations in convalescent patients [7,8]. Not only detecting ventricular dysfunction but confirming the presence of fibrosis and oedema as well, that was detectable in 26–60% of patients. However, a number of the reviewed studies contained a large spectrum of disease severity in the acute phase and were not limited to the mildest of cases. Studies with predominantly mild disease severity found significantly less severe cardiac involvement, some interesting results actually showing no significant alterations at 6 months after asymptomatic-mild infections.

## 5. Study Limitations

The study was conducted during the COVID pandemic with restricted medical resources and limited possibilities to perform serial patient visits. As a result of this, adequate control group was possible to be recruited well after the study population was assessed. In addition, serial echocardiographic measurements were not possible to perform in order to follow the time-course of the alterations detected in the patients.

## 6. Conclusions

- During the post-acute phase of even mild COVID-19 subtle functional alterations can be detected by extended echocardiographic protocols including advanced deformation imaging;
- Although altered echocardiographic parameters may include traditional echocardiographic parameters (e.g., LV ejection fraction, LV end diastolic diameter, etc.), their relative change is generally modest;
- Along with non-invasive measurement of stroke volume, deformation imaging appears to be able to detect the most pronounced relative difference for both left and right ventricular function, with left ventricular global myocardial work index and right ventricular free wall strain being the most robust alteration;
- These minor changes are difficult to utilize on a single patient basis, however, LV myocardial work and RV free wall strain seem to be the most sensitive and reproducible 2D echo-based functional parameters for screening for cardiac injury and follow-up;
- Echocardiography, even advanced investigations requiring expert echocardiog-raphers, is less time consuming and more available than cardiac magnetic resonance imaging, and thus more suited for larger scale screening and follow-up for myocardial injury after COVID-19 infection;
- Despite widespread vaccination has reduced the severity of subsequent waves of the pandemic, it is expected to remain a global health issue, and thus further studies and ongoing research into the cardiac involvement of the disease is warranted.

## Figures and Tables

**Table 1:** Baseline clinical characteristics of study patients.

	Control Group (*n* = 60)	Post-COVID Group (*n* = 86)	Relative Difference (%) ^†^
age, year	40.3 ± 11.0	39.5 ± 13.0	NA
male sex	24 (40.0)	30 (34.9)	NA
BSA, m^2^	1.9 ± 0.3	1.9 ± 0.3	NA
systolic blood pressure, Hgmm	130.3 ± 12.8	132.2 ± 15.8	NA
diastolic blood pressure, Hgmm	75.0 (67.0–82.0)	78.0 (67.8–86.0)	NA
previously treated/diagnosed hypertension	7 (11.7)	10 (11.8)	NA
**heart rate, bpm**	**70.9 ± 10.8**	75.6 ± 13.4 *	**9.5**

Values are given as mean ± SD, median (interquartile range) or *n* (%). Values are considered statistically significantly different at *p* < 0.05 (*), compared with the control group. Significant differences are marked with asterisk and printed in bold. ^†^ Relative difference is given only for parameters showing statistical difference compared to controls. BSA: body surface area; NA: not applicable.

**Table 2:** Echocardiographic parameters of the left atrium and left ventricle in the study groups.

	Control Group (*n* = 60)	Post-COVID Group (*n* = 86)	Relative Difference (%) †
left atrial volume, mL	50.0 (42.3–60.8)	50.0 (40.0–60.0)	NA
left atrial volume index, mL/m^2^	26.0 (23.0–31.0)	27.0 (22.0–32.0)	NA
**left ventricular end diastolic diameter, mm**	**46.2 ± 4.2**	47.9 ± 4.2 *	**3.2**
left ventricular end systolic diameter, mm	30.0 (27.5–33.0)	30.0 (27.0–33.0)	NA
left ventricular end diastolic volume, mL	91.5 (72.0–118.5)	97.0 (81.0–114.0)	NA
left ventricular end diastolic volume index, mL/m^2^	49.5 ± 11.2	51.9 ± 12.8	NA
left ventricular end systolic volume, mL	32.0 (24.0–36.5)	32.5 (27.0–41.0)	NA
**left ventricular end systolic volume index, mL/m^2^**	**15.5 (13.3–18.6)**	17.1 (15.2–21.0) *	**9.9**
interventricular septum, mm	9.0 (8.0–9.5)	9.0 (8.0–10.0)	NA
**posterior wall, mm**	**8.5 (8.0–9.0)**	9.0 (8.0–10.0) *	**5.9**

Values are given as mean ± SD, median (interquartile range) or *n* (%). Values are considered statistically significantly different at *p* < 0.05 (*), compared with the control group. Significant differences are marked with asterisk and printed in bold. † Relative difference is given only for parameters showing statistical difference compared to controls.

**Table 3:** Echocardiographic parameters of the systolic and diastolic function of the left atrium and left ventricle in the study groups.

	Control Group (*n* = 60)	Post-COVID Group (*n* = 86)	Relative Difference (%) †
**LV ejection fraction, %**	**68.0 (65.0–70.0)**	66.0 (60.0–70.0) *	**2.9**
LVOT velocity time integral, cm	23.0 (21.2–24.5)	22.2 (20.2–24.9)	NA
**LV stroke volume, mL**	**75.5 (70.0–87.0)**	70.5 (61.0–78.0) **	**6.6**
**LV stroke volume index, mL/m^2^**	**41.6 (38.9–43.7)**	37.4 (33.5–41.8) ***	**10.0**
LV cardiac output, L/min	5.5 ± 1.1	5.4 ± 1.2	NA
LV cardiac index, L/min/m^2^	2.9 ± 0.5	2.9 ± 0.6	NA
**LV global longitudinal strain, %**	**−20.3 (−21.1–−19.0)**	−19.1 (−20.4–−17.6) ***	**5.9**
**LV global work index, Hgmm%**	**1975 (1789–2105)**	1829 (1656–2057) **	**7.4**
LV global constructive work, Hgmm%	2383 (2226–2577)	2341 (2094–2559)	NA
LV global wasted work, Hgmm%	99 (63–129)	107 (77–151)	NA
**LV global work efficiency, %**	**96 (94–97)**	95 (93–96) *	**1.0**
transmitral E velocity, cm/s	82.0 ± 13.5	82.21 ± 15.7	NA
transmitral A velocity, cm/s	59.0 (51.3–70.5)	61.0 (54.0–76.0)	NA
E/A	1.35 (1.15–1.63)	1.31 (1.07–1.63)	NA
mitral annulus e’ velocity, cm/s	14.5 (12.0–16.0)	13.0 (11.0–17.0)	NA
mitral annulus a’ velocity, cm/s	9.0 (8.0–12−0)	10.0 (8.0–12.0)	NA
mitral annulus s’ velocity, cm/s	11.0 (10.0–13.0)	10.0 (9.0–12.0)	NA
E/e’	5.6 (4.9–6.8)	6.0 (5.2–7.3)	NA

Values are given as mean ± SD, median (interquartile range) or *n* (%). Values are considered statistically significantly different at *p* < 0.05 (*), *p* < 0.01 (**), *p* < 0.001 (***), compared with the control group. Significant differences are marked with asterisk and printed in bold. † Relative difference is given only for parameters showing statistical difference compared to controls. LV: left ventricle; LVOT: LV outflow tract, LA: left atrium.

**Table 4:** Echocardiographic parameters of dimension and function of the right atria and right ventricle in the study patients.

	Control Group (*n* = 60)	Post-COVID Group (*n* = 86)	Relative Difference (%) †
right atrial area, cm^2^	14.0 (11.0–16.4)	14.0 (12.0–16.7)	NA
right ventricular basal diameter, mm	35.0 ± 4.5	35.6 ± 5.6	NA
right ventricular diameter at the level of the papillary muscles, mm	29.0 ± 5.1	29.7 ± 4.7	NA
**tricuspid annular plane systolic excursion, mm**	**23.75 ± 2.8**	22.5 ± 3.4 *	**5.3**
tricuspid annular s’ velocity, mm	14.0 (13.0–15.0)	13.0 (12.0–15.0)	NA
**right ventricular free wall strain, %**	**−26.6 ± 3.80**	−23.8 ± 4.0 ***	**11.7**

Values are given as mean ± SD, median (interquartile range) or *n* (%). Values are considered statistically significantly different at *p* < 0.05 (*), *p* < 0.001 (***), compared with the control group. Significant differences are marked with asterisk and printed in bold. † Relative difference is given only for parameters showing statistical difference compared to controls.

**Table 5:** Univariate and multivariate correlation analysis of advanced echocardiographic parameters.

	GWI		GWE		GLS	
	Univariate Correlation	Multivariate Correlation	Univariate Correlation	Multivariate Correlation	Univariate Correlation	Multivariate Correlation
LV ejection fraction, %	0.220 *	NS	0.214 *	NS	−0.252 *	NS
LVOT velocity time integral, cm	0.336 **	NS	NS	NS	NS	NS
LV stroke volume index, mL/m^2^	0.336 **	NS	NS	NS	−0.387 ***	−0.284 *
LV cardiac index, L/min/m^2^	NS	NS	NS	NS	−0.262 *	NS
LV global longitudinal strain, %	−0.551 ****	NA	−0.561 ****	NA	NA	NA
LV global work index, Hgmm%	NA	NA	NA	NA	−0.551 ****	NA
transmitral E velocity, cm/s	NS	NS	0.326 **	NS	−0.267 *	NS
E/A	NS	NS	NS	NS	−0.252 *	NS
mitral annulus e’ velocity, cm/s	NS	NS	0.381 ***	NS	−0.328 **	NS
mitral annulus s’ velocity, cm/s	NS	NS	0.240 *	NS	−0.219 *	NS
E/e’	0.247 *	NS	NS	NS	NS	NS
left atrial diameter, medio-lateral, mm	0.334 **	NS	NS	NS	NS	NS
left atrial height, mm	0.248 *	NS	NS	NS	NS	NS
left atrial volume, mL	0.249 *	NS	0.233 *	NS	NS	NS
left atrial volume index, mL/m^2^	0.321 **	NS	0.286 **	NS	−0.263 *	−0.343 **
left ventricular end systolic volume, mL	NS	NS	NS	NS	0.242 *	NS
systolic blood pressure, Hgmm	0.614 ****	NA	NS	NS	NS	NS
diastolic blood pressure, Hgmm	0.479 ****	NA	NS	NS	NS	NS

Values represent Pearson’s or Spearman’s correlation coefficient (r), or partial r in case of multivariate analysis. Values are considered statistically significantly different at *p* < 0.05 (*), *p* < 0.01 (**), *p* < 0.001 (***), *p* < 0.0001 (****). Significant differences are marked with asterisk and printed in bold. GLS: global longitudinal strain; GWE: global work efficiency; GWI: global work index; LV: left ventricle, NS: not significant; NA: not applicable.

## Data Availability

To ensure independent interpretation of the study results, the authors grant all external authors access to relevant material, including participant-level clinical study data. Study documents and participant clinical study data are available to be shared on request after publication of the primary manuscript. Bona fide, qualified scientific and medical researchers are eligible to request access to the clinical study data with corresponding documentation describing the structure and content of the datasets. Data are shared in a secure data-access system. Prior to providing access, clinical study documents and data will be examined, and, if necessary, redacted and de-identified to protect the personal data of the study participants and personnel, and to respect the boundaries of the informed consent of the study participants.
